# Detection and molecular analysis of Pseudorabies virus from free-ranging Italian wolves (*Canis lupus italicus*) in Italy - a case report

**DOI:** 10.1186/s12917-023-03857-0

**Published:** 2024-01-03

**Authors:** Ana Moreno, Carmela Musto, Marco Gobbi, Giulia Maioli, Marika Menchetti, Tiziana Trogu, Marta Paniccià, Antonio Lavazza, Mauro Delogu

**Affiliations:** 1 Istituto Zooprofilattico Sperimentale della Lombardia e dell’Emilia-Romagna, 25124 Brescia, Italy; 2 Department of Veterinary Medical Sciences, University of Bologna, Ozzano dell’Emilia, Bologna, 40064 Italy; 3 Istituto Zooprofilattico Sperimentale dell’Umbria e delle Marche, Perugia, 06126 Italy; 4 Neurology and Neurosurgery Division, San Marco Veterinary Clinic, Veggiano, Padua 35030 Italy

**Keywords:** Aujeszky’s disease, *Canis lupus italicus*, PrV, Phylogenetic analysis, Wild boar

## Abstract

**Background:**

The only natural hosts of Pseudorabies virus (PRV) are members of the family Suidae (*Sus scrofa scrofa*). In mammals, the infection is usually fatal and typically causes serious neurologic disease. This study describes four Aujeszky’s disease cases in free-ranging Italian wolves (*Canis lupus italicus*). In Italy, the wolf is a strictly protected species and is in demographic expansion.

**Case presentation:**

Three wolves (Wolf A, B, and C) were found in a regional park in Northern Italy, and one (Wolf D) was found in Central Italy. Wolf A and D were alive at the time of the finding and exhibited a fatal infection with epileptic seizures and dyspnoea, dying after a few hours. Wolf B presented scratching lesions under the chin and a detachment of the right earlobe, whilst Wolf C was partially eaten.

The wolves showed hepatic congestion, diffuse enteritis, moderate pericardial effusion, severe bilateral pneumonia, and diffuse hyperaemia in the brain. The diagnostic examinations included virological analyses and detection of toxic molecules able to cause serious neurological signs.

All four wolves tested positive for pseudorabies virus (PrV). The analysed sequences were placed in Italian clade 1, which is divided into two subclades, “a” and “b”. The sequences of Wolf A, B, and C were closely related to other Italian sequences in the subclade b, originally obtained from wild boars and hunting dogs. The sequence from Wolf D was located within the same clade and was closely related to the French hunting dog sequences belonging to group 4.

**Conclusion:**

Results showed the presence of PrV strains currently circulating in wild boars and free-ranging Italian wolves. The genetic characterisation of the PrV UL44 sequences from the four wolves confirmed the close relationship with the sequences from wild boars and hunting dogs. This fact supports a possible epidemiological link with the high PrV presence in wild boars and the possibility of infection in wolves through consumption of infected wild boar carcasses or indirect transmission. To the best of our knowledge, this study is the first detection of Pseudorabies virus in free-ranging Italian wolves in northern and central Italy.

**Supplementary Information:**

The online version contains supplementary material available at 10.1186/s12917-023-03857-0.

## Background

Pseudorabies virus (PrV) is a herpesvirus belonging to the subfamily *Alphaherpesvirinae*, genus *Varicellovirus* that causes Aujeszky's disease (AD), characterised in swines by a variety of age-dependent clinical signs such as respiratory, reproductive, and neurological symptoms. Therefore, AD causes severe economic losses in pig farms and is categorised as a C listed disease in many European countries, according to the Commission Implementing Regulation (EU) 2018/1882 of December 3, 2018. In Europe, most countries have obtained indemnity status for AD by implementing its eradication, control, and monitoring programs based on mandatory vaccination in pigs. In Italy, vaccination against AD is compulsory except for three regions (Trentino Alto Adige, Friuli Venezia Giulia, and Veneto), which have already achieved indemnity status and therefore are included in Annex VI Part I of EU Delegated Regulation 2021/620 flagged as free from AD. In addition, three other northern Italian regions (Emilia-Romagna, Lombardy, and Piedmont) decided to undertake a unified pathway to obtain indemnity status and they have suspended vaccination against AD in all pig farms since January 2022. However, AD is continuously reported in wild boars and hunting dogs, even in many countries that have already successfully eradicated the disease in domestic pigs using large-scale vaccination programs with gE-deleted vaccines [1, 2]. Moreover, beside suids, which are the natural host of the virus [3], other wildlife and domestic animals can be infected by the virus through ingestion of raw porcine tissues or through contact with infected pigs or wild boars, resulting in neurological signs that are usually fatal within a few days. AD cases have been detected in carnivores including foxes [4], bears [5], wolves [6], raccoons [7], Iberian lynx [8], and panthers [9]. Clinical cases are also reported in domestic animals, as cattle, cats, and dogs [10, 11], and in farmed foxes and minks [12]. In addition to virulent field strains, which proved to be highly pathogenic, the attenuated PrV strains used in vaccines were also pathogenic for dogs [13], sheep [14], and lambs [15].

This case report considers four cases of mortality in wolves in two regions, Emilia-Romagna and Umbria. The Emilia-Romagna region (northern Italy) is estimated to host a considerable percentage of the whole wolf population in Italy [16], which have been expanding their distribution and numbers since the 1990s [17]. In this region, wild ungulate populations have among the highest densities in Europe and are exposed to intensive culling and recreational hunting [18]. A similar situation can be found in the Umbria region [16]. In both regions, the wolf diet is based mainly on wild ungulates (>90%) and the wild boar is the most common species in the diet [19], with greater preference for piglets, i.e., subjects with body mass class of 10-35kg [19, 20].

Serological analyses for the detection of PrV antibodies in wild boars in the province of Bologna in the period 2010-2021 showed a seroprevalence of 29.4% [21], very similar with the data from the Umbria Region, with seroprevalence of 33% (Epidemiological Surveillance of the Umbria region - unpublished data).

In this study, we describe an AD case report of three Italian wolves found in a regional park in the Emilia-Romagna region. Additionally, we included another symptomatic case from the Umbria region. To the best of our knowledge, this is the first detection of AD in free-ranging Italian wolves in northern and central Italy.

## Case presentation

### Material and methods

#### Case presentation 1

**Fig. 1:**
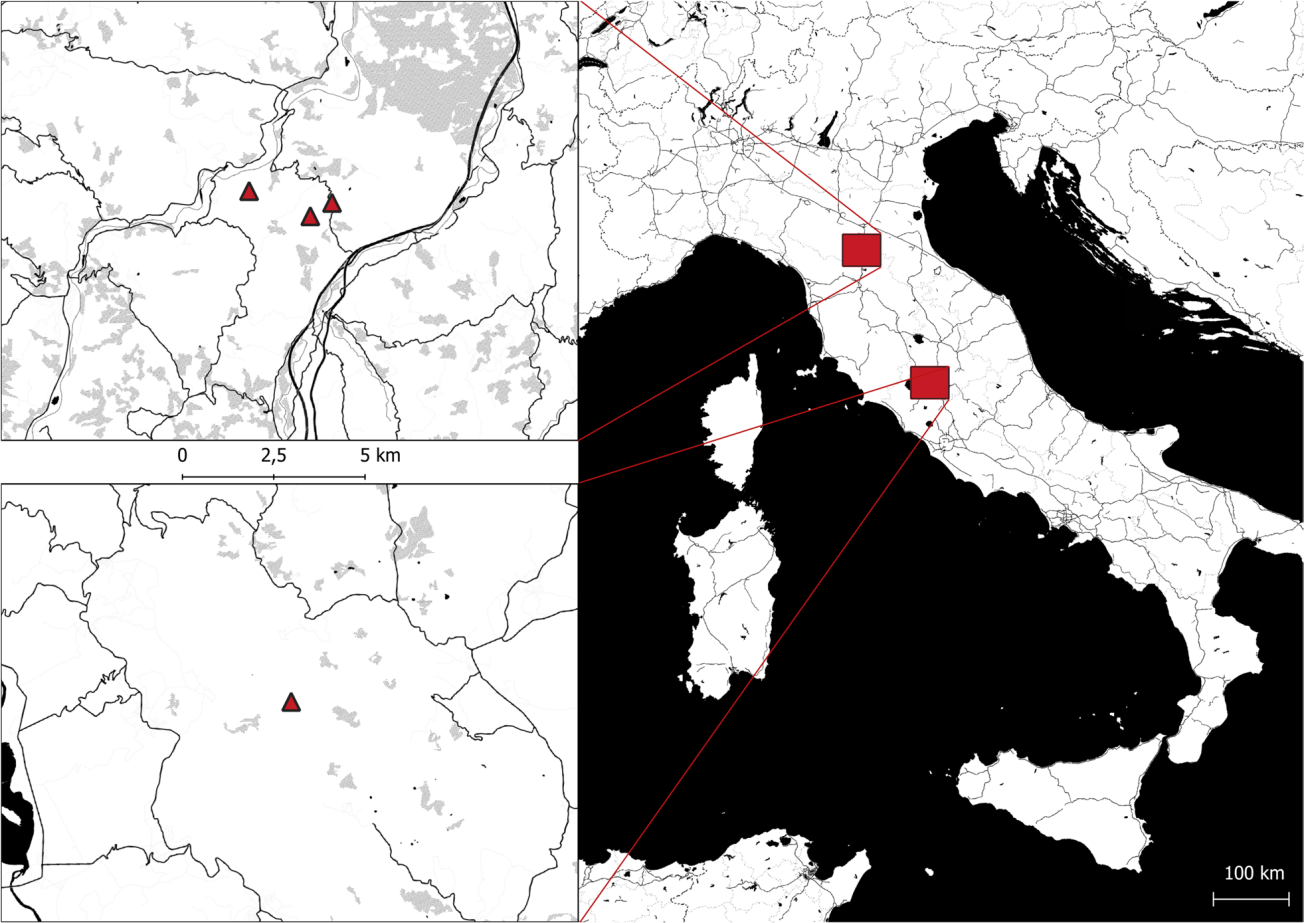
Map of collected wolves in the study area. The triangles represent the location where wolves were found. The position of the Emilia-Romagna region is in the upper left corner, while Umbria region is in the lower left corner of the figure. The map was created by Dr. Rudy Brogi using the open-source software QGIS 3.10

An adult male wolf (Wolf A) displaying dyspnoea and severe neurological signs (i.e., seizures, hyperexcitability, ataxia, severe itching) was found in January 2022 in the province of Bologna, within the area of the regional park “Monte Sole”, in northern Italy (Fig. 1). The wolf was recovered by the wildlife rescue centre “Monte Adone” where he died after 48 hours. The dead wolf was conferred on the “Istituto Zooprofilattico Sperimentale della Lombardia e dell’Emilia-Romagna – IZSLER” laboratories on the same day.

#### Case presentation 2

Two adult female wolves (Wolf B and Wolf C) were found dead in the same area (Fig. 1) and died few days after the finding of Wolf A, they were conferred on the “Department of Veterinary Medical Sciences-University of Bologna” laboratories to ascertain the cause of death. Wolf B presented detachment of the right earlobe, whilst Wolf C had been partially eaten. In detail, Wolf C presented: absence of the skin of the entire neck and of the cranial thoracic region; absence of the superficial muscles of the neck (rhomboid, splenius, superficial trapezius, dentate of the neck, and thoracic trapezius); detachment of the right earlobe; finally, partial consumption of the right temporal muscle.

#### Case presentation 3

A fourth adult male wolf (Wolf D) was found in November 2022 in the province of Terni near the Natural reserve of Alviano **(**Fig. 1**)** in the Umbria region and, according to the local authorities it had been probably poisoned. The animal was found in a condition of possible status epilepticus, with altered mental status, dyspnoea, and dense salivation in the buccal rim (possibly a final phase of a previous epileptic seizure), followed by a complete generalized tonic-clonic seizure with lateral recumbency, tonic-clonic movements of the four limbs, and vocalizations (Video S1). The subject spontaneously died few minutes after being rescued by the local animal rescue centre “Wild Umbria”. Wolf D was conferred on the same day on the “Istituto Zooprofilattico Sperimentale dell'Umbria e delle Marche - IZSUM” laboratories.

#### Diagnostic examinations

The animals’ age was estimated based on dental development and wear [22], body size, and weight. One was classified as sub-adult – i.e., class 2, age between 12 months to 24 months – and three subjects were classified as adults – i.e., class 3, age greater than 2 years.

All wolves underwent necropsy during which tissue collection was performed. Before proceeding with organ sampling, each organ was inspected and assessed individually.

The brain, duodenum, tongue, muscle, popliteal lymph nodes, liver, spleen, lung, bladder, and stomach contents were collected for virological, parasitological, and toxicological examinations. The spleen, liver, kidney, lung, heart, thymus, and mediastinal lymph nodes were collected for histological examinations.

Several diagnostic tests were performed by IZSLER and IZSUM to identify the cause of the neurological disorder and death. Differential diagnosis included rabies virus, canine distemper virus (CDV), pseudorabies virus (PrV), and canine parvovirus (CPV). In addition, other diagnostic tests – in the context of the regional passive surveillance program – were conducted to investigate the health status and the presence of zoonotic parasites. The parasitic diagnosis consisted in the detection of *Trichinella* larvae and *Leishmania infantum*, performed, respectively, by artificial digestion of the muscle [23] and by real-time PCR [24] in the spleen and popliteal lymph nodes.

Virological analyses included detection of rabies carried out by direct immunofluorescence in the brain samples [25], CDV in the lung and bladder [26], and CPV in the duodenum and tongue [27], performed using different PCRs [28, 29] and brain for PrV [10].

In all four subjects, the stomach contents and liver were also analysed to detect the presence of toxic substances (i.e., zinc phosphide, strychnine, organophosphate pesticides, metaldehyde, and anticoagulants) according to methods of Bertero et al., 2020 [30].

#### Sequencing and phylogenetic analysis

The UL44 and US8 genes respectively encoding the glycoproteins gC and gE were partially sequenced from brain tissues as previously described [10]. Briefly, DNA was extracted using the QIAsymphony instrument and then amplified using primers and the PCR protocol described by Fonseca et al. (2010) [31]. The PCR products were purified using a QIAquick Gel Extraction Kit (Qiagen, Inc., Valencia; CA, USA). DNA sequencing was performed using a BigDye Terminator Cycle Sequencing Kit (Applied Biosystems, Foster City, CA, USA) with the same primers that were used for the amplification. Sequences were edited using the SeqMan software (DNASTAR, Madison®, USA) and were compared with reference sequences and wild-type PrV strains available in GenBank for phylogenetic analysis. Phylogenetic trees were constructed using the maximum likelihood (ML) method within the IQ-TREE software [32] with bootstrap analyses involving 1000 replicates. Sequence alignments were performed using the ClustalW method (DNASTAR, Madison, USA) and were manually optimised. The best-fit model for nucleotide substitution was determined with ModelFinder [33] resulting in the best-fit model HKY+F+I (according to BIC) for both gC and gE sequence datasets. The topologies were verified by the Neighbor Joining method and Kimura's two-parameter model using MEGA version 11 [34].

## Results

### Examination case 1

During the post-mortem examinations, one of wolves that was found alive (Wolf A) revealed good nutritional and hydration status. The pathological examination found hepatomegaly, hepatic congestion, increased reactivity of intestinal lymphoid tissue, severe bilateral pneumonia with oedema and lung hepatization (specifically, in the apical lobe, the middle lobe, and partly in the caudal one), and with foam in the tracheal lumen. Furthermore, there was a clinical picture of diffuse vasculitis, even in the subcutis that appeared strongly congested.

The histology examination showed: in the spleen, chronic, moderate, and diffuse follicular lymphoid depletion with centre-follicular hyalinosis; in the thymus, acute, severe, and diffuse hyperaemia with parenchymal oedema and multifocal cellular karyorrhexis; in the mediastinal lymph nodes, acute, severe, and diffuse blood resorption; in the liver, massive, acute, mild, and diffuse hydropic degeneration of hepatocytes with hyperaemia and sinusoidal leucocytosis; in the kidneys, moderate and diffuse cortical and medullary hyperaemia; in the lungs: acute, severe, and diffuse fibrinopurulent bronchopneumonia with hyperaemia, and interlobular oedema. A parasitic elongated oval egg of about 75 x 35 micrometres, with prominent asymmetrical poles and characterized by a thin hyaline brownish shell (compatible with egg of *Capillaria spp*.) was observed in the bronchioles. The heart did not show relevant findings. See Table S1.

### Examination case 2

During the post-mortem examinations, the two wolves that were found dead, Wolf B and Wolf C, revealed poor nutritional status and severe dehydration. They were frozen at -20°C, which prevented the histological investigation from happening, but the macroscopic pathological examination found strongly congested organs, as well as: gastritis, diffuse enteritis, moderate pericardial effusion, severe bilateral pneumonia, and diffuse hyperaemia in the brain. During the skinning of the subjects, the vascular pattern was noteworthily evident throughout the subcutis, a finding compatible with a suspect cutaneous vasculitis. Wolf B presented detachment of the right earlobe and subcutaneous hematomas in the chin region, findings compatible with strong scratching. It (Wolf B) also presented perianal soiling, suggesting a diarrheic status before death. See Table S1.

### Examination case 3

During the post-mortem examinations, the second wolf that was found alive (Wolf D) revealed good nutritional and hydration status. Moreover, there were no signs of scratching at the subcutis level. Macroscopic pathological examination found diffuse congestion in many organs: in the thoracic cavity, the lungs were heavily congested with also moderate serohemorrhagic exudate in the pleural cavity; in the abdominal cavity, there was diffuse congestion of the spleen, liver, and kidneys. There was also catarrhal gastritis and diffuse enteritis. The subject was stored at -20°C, therefore it was not possible to carry out a histology examination. See Table S1.

The search for toxic molecules that could justify a severe neurological disorder gave negative results. Wolf B and Wolf C tested positive for the latest generation of anticoagulants (*Bromadiolone* and *Brodifacoum*), at very reduced concentrations (Table S1).

Each wolf was negative for detection of *Trichinella* and *Leishmania infantum*. All virological tests were negative except for the presence of PrV in all subjects (Table S1).

### Sequencing and phylogenetic results

Positive PrV samples were identified as Wolf/Italy/91927/2022, Wolf/Italy/127601-2/2022, Wolf/Italy/127602-3/2022, and Wolf/Italy/7924/2023. The last one was obtained from a dead animal in December 2022, but was submitted for virus isolation and sequencing in 2023. The partial sequences of the UL44 and US8 genes of the three Italian samples have been deposited in NCBI GenBank with the accession numbers: UL44 Wolf/Italy/91927/2022- OR234026, UL44 Wolf/Italy/127601-2/2022 - OR234025, UL44 Wolf/Italy/127602-3/2022- OR234027, and UL44 Wolf/Italy/7924/2023- OR234024; US8 Wolf/Italy/91927/2022- OR234030, US8 Wolf/Italy/127601-2/2022- OR234029, US8 Wolf/Italy/127602-3/2022- OR234031, and US8 Wolf/Italy/7924/2023- OR234028.

The phylogenetic analysis of the sequences of the four Italian wolves was based on the partial sequencing of the UL44 (648 bp) and US8 (403 bp) genes encoding the gC and gE proteins and was performed by comparison with other field and reference PrV sequences.

**Fig. 2:**
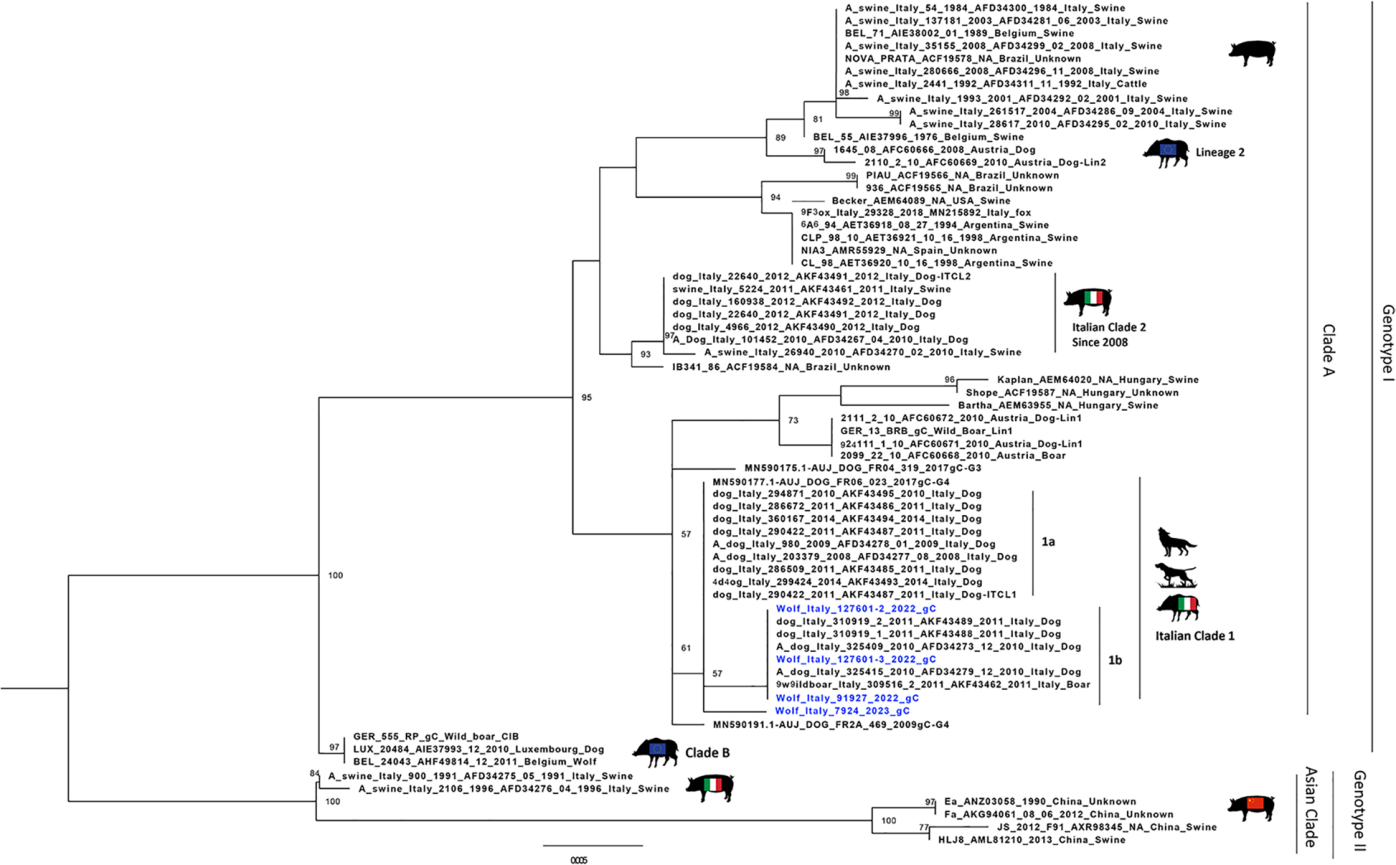
Maximum likelihood phylogenetic tree based on the partial gC gene. Phylogenetic tree was performed using the IQtree software (v 1.6.12) and the best-fit model according to BIC (HKY+F+I). The Italian PrV sequences originated from the wolves (evidenced in blue) were compared with other field and references PrV sequences. Sequences are identified with protocol number, accession no., host, and country

Only two wolf UL44 sequences were available in GenBank, one from Belgium in 2011 and the other from southern Italy in 2018. The latter was not included in the analysis because the available sequence was shorter than those included in our dataset. The phylogenetic tree of the UL44 gene (Fig. 2) showed the presence of two genotypes, I and II.

**Fig. 3:**
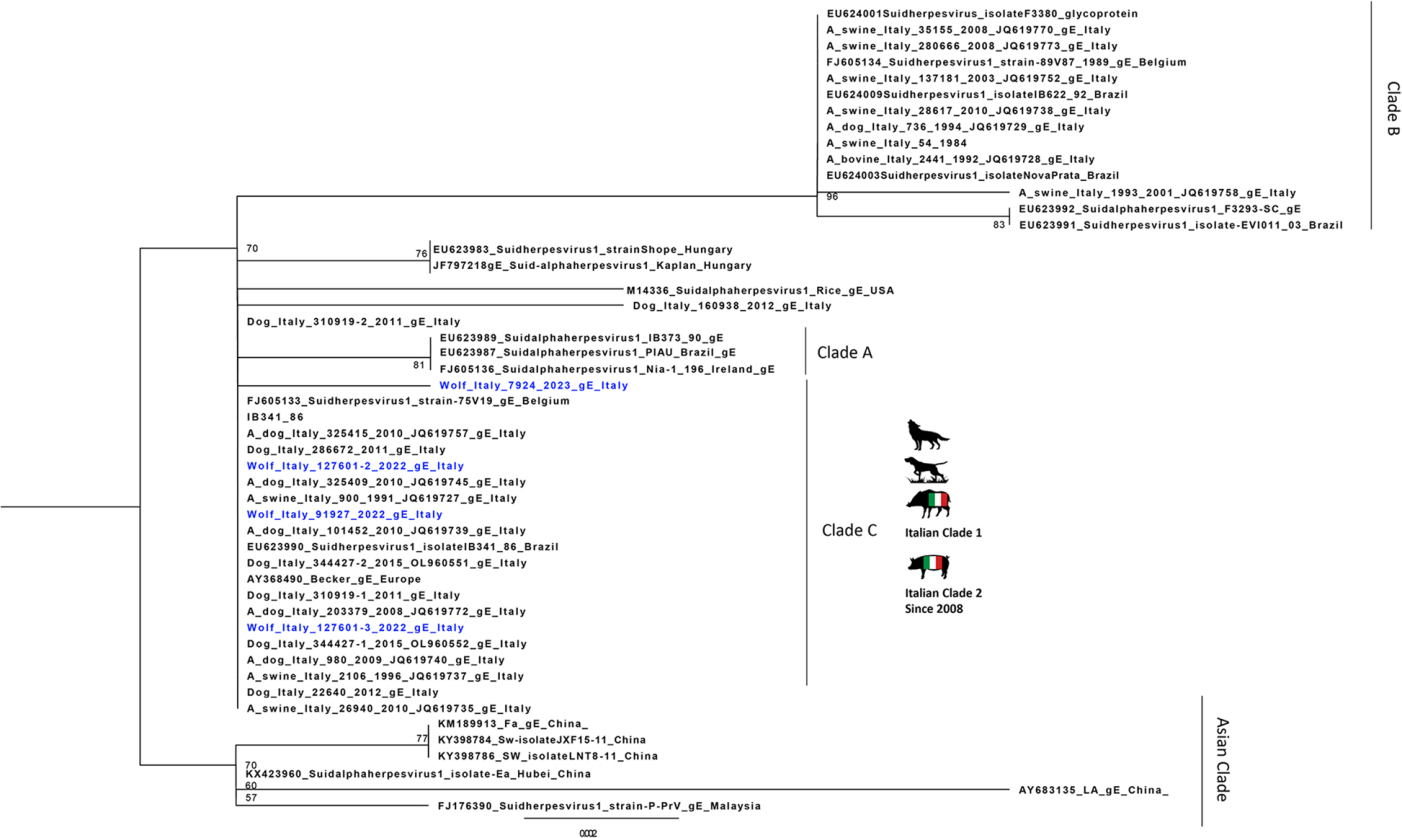
Maximum likelihood phylogenetic tree based on the partial gE gene. Phylogenetic tree was performed using the IQtree software (v 1.6.12) and the best-fit model according to BIC (HKY+F+I). The Italian PrV sequences originated from the wolves (evidenced in blue) were compared with other field and references PrV sequences. Sequences are identified with protocol number, accession no., host, and country

The US8 gene encoding the gE protein was found to be a highly conserved gene and therefore much less informative than the UL44 gene. Thus, the low number of informative sites led to a phylogenetic tree with not very high bootstrap values. This, together with the smaller number of US8 sequences from Europe available in GenBank, makes its phylogenetic analysis less revealing. The phylogenetic tree revealed the presence of four clades, named A, B, C, and Asia, as reported in the study by Fonseca et al. (2010) [31] (Fig. 3).

The analysis of deduced amino acid (aa) sequences of the UL44 gene revealed two different patterns in the sequences of the four Italian wolves. The first three sequences showed patterns of aa deletions or insertions of Italian clade 1, characterised by a single aa deletion in position 25 and the profile (VVV-E) related to the Italian wild boar strains in the hotspot region located between residues 180 and 185. The remaining sequence showed a profile that differed only in the hotspot region (VVVDD), which was identical to the French sequence AUJ/DOG/FR04/319/2017 (Fig. S1).

## Discussion and conclusion

This is the first report of AD in free-ranging wolves caused by PrV strains with an epidemiological link attributable to the high seroprevalence (29.4% and 33%) in the wild boar population persisting in the study area.

The various pathological findings mainly involved the respiratory [35, 36] and digestive [4, 9, 37] systems. These pathological findings have been confirmed in wolves [38] and in other species with pseudorabies infection [4, 8, 9, 35, 36].

All other virological (Rabies, CPV2, CDV) and parasitological (Trichinella and Leishmania) investigations were negative. Two wolves (Wolf B and Wolf C) tested positive for anticoagulants (ARs), Brodifacoum and Bromadiolone, but the traces detected in the livers indicated low concentrations, unlikely to cause an acute state of intoxication [39].

The genetic characterisation of the PrV UL44 sequences from the four wolves confirms the close relationship with the sequences from wild boar and hunting dogs, which are different from those from pigs. This fact supports a possible epidemiological link with the high PrV presence in wild boars and the possibility of infection in wolves through consumption of infected wild boar carcasses or indirect transmission. The phylogenetic tree of the UL44 gene showed the presence of two genotypes, I and II.

Genotype I of the PrV group includes sequences from Europe and the Americas, while genotype II includes only the Asian clade with sequences mainly from China. The Italian strains all belong to clade A, except for three strains isolated in the 1990s belonging to the Asian clade [40]. PrV sequences detected in Italy in the last 15 years are classified into two distinct clades based on the origin of the sequences, whether the samples come from domestic pigs or farm dogs (Italian clade 2), or from wild boars or hunting dogs (Italian clade 1), as previously published in Moreno et al. [10]. Italian clade 1 can be further divided into two groups called “a” and “b”. It is not surprising that the sequences originating from the four Italian wolves in this study were placed within Italian clade 1, which correlates with sequences originating from hunting dogs and wild boars. However, differences were observed between the three sequences from 2022 and the sequence from 2023. The NCBI blast analysis revealed that the three 2022 sequences showed 100% identity with the Italian hunting dogs’ sequences belonging to Italian clade 1b, whereas the 2023 sequence showed 99.54% identity with the AUJ/DOG/FR04/319/2017 strain [41] and 99.38% identity with the Italian clade 1a sequences. In the phylogenetic tree, the three 2022 sequences were placed in Italian clade 1b as expected, while the 2023 sequence was placed in a single branch within Italian clade 1.

It is worth noting that all the Italian US8 sequences obtained in the last 15 years, from samples whose UL44 sequences belonged to the three distinct clades (Italian clade 1, 2 and Asian clade), were all placed together in clade C in the gE phylogenetic tree. The sequences of the four wolves in this study were also placed in clade C, despite the difference observed in the UL44 gene between the 2022 and 2023 sequences. Interestingly, this clade was previously reported [31] as a new clade including a single strain isolated in Brazil in 1986.

The analysis of deduced amino acid (aa) sequences of the UL44 gene revealed two different patterns in the sequences of the four Italian wolves. The first three sequences showed patterns of aa deletions or insertions of Italian clade 1, characterised by a single aa deletion in position 25 and the profile (VVV-E) related to the Italian wild boar strains in the hotspot region located between residues 180 and 185. The remaining sequence showed a profile that differed only in the hotspot region (VVVDD), which was identical to the French sequence AUJ/DOG/FR04/319/2017. This region showed the greatest variation in aa and was associated with changes in the hydrophobicity profile [42].

These authors support the theory that Aujeszky's disease – given the high prevalence in wild boars, its wild host – is underdiagnosed in grey wolves and other wild carnivores.

Supporting this theory there are two aspects: (*i*) the fact that grey wolves base a large part of their diet on wild boars [19, 20], in particular piglets – a vulnerable weight class, i.e. from 10 to 35 kg [19] – probably because they have fewer possibilities than adults to defend themselves; (*ii*) the class selected by wolves coincides with the age class in which the virus is most successful in causing viraemia. Pigs exhibit a pronounced age resistance against PrV, with younger animals being more susceptible to fatal infections, characterized by neuronal signs, e.g., ataxia, convulsions, and sudden death. In contrast, older animals (>1 year) primarily present respiratory distress or even subclinical infection [43]. The predatory selection on the age group susceptible to the virus, associated with the high seroprevalence found in the wild reservoir, could suggest that the circulation of PrV in wild carnivores is higher than previously found.

Even if infection via ingestion appears the most plausible, it should be noted that indirect transmission can also occur through viral excretion from pigs, without direct contact with the pigs themselves [44].

Collecting wolves’ carcasses can be challenging, especially in case of natural mortality or in case of death in areas difficult to reach [45], and, moreover, not all dying or dead wolves are found. These difficulties are limiting, as they can generate statistical and methodological bias, preventing from inferring the data on the national population [45, 46]. However, for studies on elusive species such as large carnivores, the finding of carcasses is often the only opportunity of collecting samples for research, albeit maintaining a precautionary approach in interpreting the data [47].

Due to the present findings, the detection of PrV in gray wolves is now included in the regional epidemiological surveillance, in addition to wild boars’ serosurveillance since 2010. We encourage the testing of deceased wolves for *Suid alphaherpesvirus 1*, especially in the presence of neurological symptoms, pulmonary pathological signs (e.g., pneumonia, hyperaemia, oedema), and/or enteric and widespread vascular congestion.

Although the true impact of PrV on wild populations appears to be unknown, the detection of PRV in four dead Italian wolves suggests that the virus could have a negative influence on free-range populations. The Italian wolf is a species classified as "Near Threatened (NT)" by the IUCN [48] and the circulation of a highly lethal virus such as PrV could compromise the conservation of the species. In the wild, the effectiveness of using attenuated live vaccine for oral immunisation of wild boars against PrV has been demonstrated [49], though the safety of this technique needs to be better studied [6].

These results underline the importance of virological surveillance in wildlife populations and domestic pigs, as well as the importance of biosecurity measures in pig farms, in order to provide insights for a better assessment of current AD eradication approaches.

### Supplementary Information

**Additional file 1:** **Video S1**: in the video (© Riccardo Mattea) we can see “Wolf D”; the animal was in a condition of possible status epilepticus, with altered mental status, lateral recumbency, dyspnoea, dense salivation in the buccal rim (possibly a final phase of a previous epileptic seizure) followed by a complete generalized tonic-clonic seizure with lateral recumbency, tonic-clonic movements of the four limbs and vocalizations. The subject spontaneously died few minutes after the video.

**Additional file 2:** **Table S1:** Summary table of body condition, age class, pathology, histopathology, virology, and toxicology for each wolf examined.

**Additional file 3:** **Fig. S1. **Amino acid alignment of gC sequences of the Italian wolf samples that were compared with representative sequences of the Italian clade 1 and 2 and reference sequences obtained from wild boars, dogs and wolf originated from Europe and Asia.

## Data Availability

The partial sequences of the UL44 and US8 genes of the three Italian samples have been deposited in NCBI GenBank with the accession numbers: UL44 Wolf/Italy/91927/2022 - OR234026, UL44 Wolf/Italy/127601-2/2022 - OR234025, UL44 Wolf/Italy/127602-3/2022 - OR234027, and UL44 Wolf/Italy/7924/2023 - OR234024; US8 Wolf/Italy/91927/2022 - OR234030, US8 Wolf/Italy/127601-2/2022 - OR234029, US8 Wolf/Italy/127602-3/2022 - OR234031, and US8 Wolf/Italy/7924/2023 - OR234028.

## Abbreviations

AD: Aujeszky’s Disease
CDV: Canine Distemper Virus
DNA: Deoxyribonucleic Acid
ML: Maximum likelihood method
QGIS: Open-Source Geographic Information System
PCR: Polymerase Chain Reaction
PrV: Pseudorabies Virus

## References

[1] Steinrigl A, Revilla-Fernández S, Kolodziejek J, Wodak E, Bagó Z, Nowotny N, Schmoll F, Ko ¨ fer J. Detection and molecular characterization of Suid herpesvirus type 1 in Austrian wild boar and hunting dogs. Vet Microbiol. 2012;157(3–4):276–284. 10.1016/j.vetmic.2011.12.033 22264387

[2] Muller T, Klupp BG, Freuling C, Hoffmann B, Mojcicz M, Capua I, Palfi V, Toma B, Lutz W, Ruiz-fon F, Gortarzar C, Hlinak A, Schaarschmidt U, Zimmer K, Coraths FJ, Hahn EC, Mettenleiter TC (2010). Characterization of pseudorabies virus of wild boar origin from Europe. Epidemiol Infect.

[3] Kluge JP, Beran GW, Hill HT, Platt KB, Leman AD, Straw BE, Mengeling WL, D’Allaire S, Taylor DJ (1992). Pseudorabies (Aujeszky’s Disease). Diseases of Swine.

[4] Moreno A, Chiapponi C, Sozzi E, Morelli A, Silenzi V, Gobbi M, Lavazza A, Paniccià M (2020). Detection of a gE-deleted Pseudorabies virus strain in an Italian red fox. Vet Microbiol.

[5] Zanin E, Capua I, Casaccia C, Zuin A, Moresco A (1997). Isolation and characterization of Aujeszky’s disease virus in captive brown bears from Italy. J Wildl Dis.

[6] Verpoest S, Cay AB, Bertrand O, Saulmont M, De Regge N (2014). Isolation and characterization of pseudorabies virus from a wolf (*Canis lupus*) from Belgium. Eur J Wildl Res.

[7] Thawley DG, Wright JC (1982). Pseudorabies virus infection in raccoons: a review. J Wildl Dis.

[8] Masot AJ, Gil M, Risco D, Jiménez OM, Núñez JI, Redondo E (2017). Pseudorabies virus infection (Aujeszky’s disease) in an Iberian lynx (*Lynx pardinus*) in Spain: a case report. J BMC Vet Res.

[9] Glass MC, McLean RG, Katz JB, Maehr DS, Cropp CB, Kirk LJ, McKeirnan AJ, Evermann JF (1994). Isolation of pseudorabies (Aujeszky’s disease) virus from a Florida panther. J Wildl Dis.

[10] Moreno A, Sozzi E, Grilli G, Gibelli LR, Gelmetti D, Lelli D, Chiari M, Prati P, Alborali L, Boniotti MB, Lavazza A, Cordioli P (2015). Detection and molecular analysis of Pseudorabies virus strains isolated from dogs and a wild boar in Italy. Vet Microbiol.

[11] Pensaert MB, Kluge P, Pensaert MB (1989). Pseudorabies virus (Aujeszky’s disease). Virus Infections of Porcines.

[12] Jin HL, Gao SM, Liu Y, Zhang SF, Hu RL (2016). Pseudorabies in farmed foxes fed pig offal in Shandong province China. Arch Virol.

[13] Lin W, Shao Y, Tan C, Shen Y, Zhang X, Xiao J, Wu Y, He L, Shao G, Han M, Wang H, Ma J, Xie Q (2019). Commercial vaccine against pseudorabies virus: a hidden health risk for dogs. Vet Microbiol.

[14] Kong H, Zhang K, Liu Y, Shang Y, Wu B, Liu X (2013). Attenuated live vaccine (Bartha-K16) caused pseudorabies (Aujeszky’s disease) in sheep. Vet Res Comm.

[15] Clark LK, Molitor TW, Gunther R, Joo HS (1984). Pathogenicity of a modified-live pseudorabies vaccine virus in lambs. J Am Vet Med Ass.

[16] Galaverni M, Caniglia R, Fabbri E, Milanesi P, Randi E (2016). One, no one, or one hundred thousand: how many wolves are there currently in Italy?. Mammal Res.

[17] Caniglia R, Fabbri E, Galaverni M, Milanesi P, Randi E (2014). Noninvasive sampling and genetic variability, pack structure, and dynamics in an expanding wolf population. J Mammal.

[18] Regione Emilia-Romagna. 2018. Piano Faunistico Venatorio Regionale 2018–2023. https://agricoltura.regione.emilia-romagna.it/caccia/temi/normativa/indirizzi-pianificazione/piano-faunistico-2018.

[19] Bassi E, Canu A, Firmo I, Mattioli L, Scandura M, Apollonio M (2017). Trophic overlap between wolves and free-ranging wolf dog hybrids in the Apennine Mountains Italy. Glob Ecol Conserv.

[20] Bassi E, Gazzola A, Bongi P, Scandura M, Apollonio M (2020). Relative impact of human harvest and wolf predation on two ungulate species in Central Italy. Ecol Res.

[21] Rossi A, Santi A, Barsi F, Casadei G, Di Donato A, Fontana MC, Galletti G, Garbarino CA, Lombardini A, Musto C (2023). Eleven Years of Health Monitoring in Wild Boars (*Sus scrofa*) in the Emilia-Romagna Region (Italy). Animals.

[22] Brasington TJ, Hadley JM, Stahler DR, Stahler EE, Cassidy KA. A visual guide to wolf dentition and age determination. Wildlife Biology 2023;1-27. https://www.researchgate.net/publication/374921532_A_Visual_Guide_to_Wolf_Dentition_and_Age_Determination_For_Researchers_and_Wildlife_Professionals.

[23] European Commission. Commission Regulation (EC) No 2075/2005 of 5 December 2005 laying down specific rules on official controls for *Trichinella* in meat (Text with EEA relevance) Off. J., L338 (2005), 68-82.

[24] Taddei R, Bregoli A, Galletti G, Carra E, Fiorentini L, Fontana MC, Frasnelli M, Musto C, Pupillo G, Reggiani A, Santi A, Rossi A, Tamba M, Calzolari M, Rugna G (2022). Wildlife Hosts of *Leishmania Infantum* in a Re-Emerging Focus of Human Leishmaniasis, in Emilia-Romagna Northeast Italy. Pathogens.

[25] OIE. Rabies. In: Manual of diagnostic tests and vaccines for terrestrial animals. Paris; 2011. p. 2.1.13. https://www.woah.org/fileadmin/Home/eng/Health_standards/tahm/A_summry.htm.

[26] Headley A, Sukura S, Antti (2009). Naturally occurring systemic canine distemper virus infection in a pup. Braz J Vet Pathol..

[27] Balboni A, Urbani L, Delogu M, Musto C, Fontana MC, Merialdi G, Lucifora G, Terrusi A, Dondi F, Battilani M (2021). Integrated Use of Molecular Techniques to Detect and Genetically Characterise DNA Viruses in Italian Wolves (*Canis lupus italicus*). Animals.

[28] Frisk AL, König M, Moritz A, Baumgärtner W (1999). Detection of canine distemper virus nucleoprotein RNA by reverse transcription-PCR using serum, whole blood, and cerebrospinal fluid from dogs with distemper. J Clin Microbiol.

[29] Decaro N, Elia G, Campolo M, Desario C, Lucente MS, Bellacicco AL, Buonavoglia C (2005). New approaches for the molecular characterization of canine parvovirus type 2 strains. J Vet Med B Infect Dis Vet Public Health.

[30] Bertero A, Chiari M, Vitale N, Zanoni M, Faggionato E, Biancardi A, Caloni F (2020). Types of pesticides involved in domestic and wild animal poisoning in Italy. Sci Total Environ.

[31] Fonseca AA Jr, Camargos MF, de Oliveira AM, Ciacci-Zanella JR, Patrício MA, Braga AC, Cunha ES, D’Ambros R, Heinemann MB, Leite RC, dos Reis JK. Molecular epidemiology of Brazilian pseudorabies viral isolates. Vet Microbiol. 2010;141(3–4):238–45. 10.1016/j.vetmic.2009.09.018 19828266

[32] Nguyen L, Schmidt HA, von Haeseler A, Minh BQ (2015). IQ-TREE: A fast and effective stochastic algorithm for estimating maximum likelihood phylogenies. Mol Biol Evol.

[33] Kalyaanamoorthy S, Minh BQ, Wong TKF, von Haeseler A, Jermiin LS (2017). Model Finder: Rapid model selection for accurate phylogenetic estimates. Nat Methods.

[34] Tamura K, Stecher G, Kumar S (2021). MEGA11: Molecular Evolutionary Genetics Analysis Version 11. Mol Biol Evol..

[35] Sehl J, Teifke JP (2020). Comparative Pathology of Pseudorabies in Different Naturally and Experimentally Infected Species—A Review. Pathogens.

[36] Kimman TG, van Oirschot JT (1986). Pathology of Aujeszky’s Disease in Mink. Vet Pathol.

[37] Sharma R, Saikumar G (2010). Porcine parvovirus- and porcine circovirus 2-associated reproductive failure and neonatal mortality in crossbred Indian pigs. Trop Anim Health Prod.

[38] Amoroso MG, Di Concilio D, D’Alessio N, Veneziano V, Galiero G, Fusco G (2020). Canine parvovirus and pseudorabies virus coinfection as a cause of death in a wolf (*Canis lupus*) from southern Italy. Vet Med Sci.

[39] Cerri J, Musto C, Capizzi D, Fontana MC, Rubini S, Merialdi G, ..., Garbarino CA. Anticoagulant rodenticides are climbing the food chain to the top: a first proof of widespread positivity in grey wolves (*Canis lupus*). Preprint, 2023, 10.32942/X2J30M.

[40] Sozzi E, Moreno A, Lelli D, Cinotti S, Alborali GL, Nigrelli A, Luppi A, Bresaola M, Catella A, Cordioli P (2014). Genomic characterization of pseudorabies virus strains isolated in Italy. Transbound Emerg Dis.

[41] Deblanc C, Oger A, Simon G, Le Potier MF (2019). Genetic Diversity among Pseudorabies Viruses Isolated from Dogs in France from 2006 to 2018. Pathogens..

[42] Fonseca AA, Camargos MF, Sales ML, Heinemann MB, Leite RC, Reis JK (2012). Pseudorabies virus can be classified into five genotypes using partial sequences of UL44. Braz J Microbiol.

[43] Mettenleiter TC. Aujeszky’s Disease and the Development of the Marker/DIVA Vaccination Concept. Pathogens. 2020;9(7):563. 10.3390/pathogens9070563 PMC7400435 32664700

[44] Thiry E, Addie D, Belák S, Boucraut-Baralon C, Egberink H, Frymus T, Gruffydd-Jones T, Hartmann K, Hosie M, Lloret A, Lutz H, Marsilio F, Möstl K, Pennisi MG, Radford AD, Truyen U, Horzinek MC (2013). Aujeszky’s disease/pseudorabies in cats. ABCD guidelines on prevention and management. J Fel Med Surg..

[45] Musto C, Cerri J, Galaverni M, Caniglia R, Fabbri E, Mucci N, Bonilauri P, Maioli G, Fontana MC, Gelmini L, Prosperi A, Rossi A, Garbarino C, Fiorentini L, Ciuti F, Berzi D, Merialdi G, Delogu M (2021). Men and wolves: are anthropogenic causes the main driver of wolf mortality in human-dominated landscapes in Italy?. Global Ecol Conserv.

[46] Musto C, Tamba M, Calzolari M, Rossi A, Grisendi A, Marzani K, Bonilauri P, Delogu M (2023). Detection of west Nile And Usutu virus RNA in Autumn Season in Wild Avian Hosts in Northern Italy. Viruses.

[47] Lovari S, Sforzi A, Scala C, Fico RA (2007). A wolf in the hand is worth two in the bush: a response to Ciucci. J Zool..

[48] IUCN (2022). Lista Rossa dei vertebrati italiani.

[49] Maresch C, Lange E, Teifke JP, Fuchs W, Klupp B, Müller T, Mettenleiter TC, Vahlenkamp TW (2012). Oral immunization of wild boar and domestic pigs with attenuated live vaccine protects against Pseudorabies virus infection. Vet Microbiol.

