# Supplementary material for: Detection and molecular analysis of Pseudorabies virus from free-ranging Italian wolves (Canis lupus italicus) in Italy - a case report
Source: BMC Vet Res. 2024 Jan 3;20:9. doi: 10.1186/s12917-023-03857-0 (PMC10765938; doi:10.1186/s12917-023-03857-0)
Supplement: Supplementary file 2 — Additional file 2: Table S1: Summary table of body condition, age class, pathology, histopathology, virology, and toxicology for each wolf examined. [file 12917_2023_3857_MOESM2_ESM.docx]

**Table S1:** Summary table of body condition, age class, pathology, histopathology, virology, and toxicology for each wolf examined.

Abbreviations: CDV - Canine Distemper Virus; CPV_2_ – Canine Parvovirus; PrV - Pseudorabies Virus

|  | **WOLF A** | **WOLF B** | **WOLF C** | **WOLF D** |
| --- | --- | --- | --- | --- |
| **BODY CONDITION** | Good | Poor | Poor (partially eaten) | Good |
| **AGE CLASS** | Sub-adult | Adult | Adult | Adult |
| **PATHOLOGY** | Hepatomegaly, hepatic congestion, increased reactivity of intestinal lymphoid tissue, pneumonia, oedema, and lung hepatization, foam in the tracheal lumen. Diffuse vasculitis, subcutis congested. | Gastritis, enteritis, pericardial effusion, bilateral pneumonia, hyperemia of the brain. Diffuse vasculitis, subcutis congested. Detachment of the right earlobe and subcutaneous hematomas in the chin region. | Gastritis, enteritis, pericardial effusion, bilateral pneumonia, hyperemia of the brain. Diffuse vasculitis, subcutis congested. | Lungs congested, moderate serohemorrhagic exudate in the pleural cavity, congestion of the spleen, liver, and kidneys. Catarrhal gastritis and diffuse enteritis. |
| **HISTOPATHOLOGY** | Spleen; chronic, moderate, and diffuse follicular lymphoid depletion with centre-follicular hyalinosis. Thymus; acute, severe, and diffuse hyperaemia with parenchymal oedema and multifocal cellular karyorrhexis.  Mediastinal lymph nodes; acute, severe, and diffuse blood resorption.  Liver; massive, acute, mild, and diffuse hydropic degeneration of hepatocytes with hyperaemia and sinusoidal leucocytosis.  Kidneys; moderate and diffuse cortical and medullary hyperaemia.  Lungs: acute, severe, and diffuse fibrinopurulent bronchopneumonia with hyperaemia, and interlobular oedema. With a parasitic elongated oval egg, compatible with egg of *Capillaria spp*. in the bronchioles.  Heart; not relevant findings. | Not performed | Not performed | Not performed |
| **VIROLOGY** | Positive; PrV Negative; Rabies, CDV, CPV_2_ | Positive; PrV Negative; Rabies, CDV, CPV_2_ | Positive; PrV Negative; Rabies, CDV, CPV_2_ | Positive; PrV Negative; Rabies, CDV, CPV_2_ |
| **TOXICOLOGY** | Negative for all compounds | Positive; ARs (*Bromadiolone, Brodifacoum*) | Positive; ARs (*Bromadiolone, Brodifacoum*) | Negative for all compounds |
