# Supplementary figures and images for: Detection and molecular analysis of Pseudorabies virus from free-ranging Italian wolves (Canis lupus italicus) in Italy - a case report
Source: BMC Vet Res. 2024 Jan 3;20:9. doi: 10.1186/s12917-023-03857-0 (PMC10765938; doi:10.1186/s12917-023-03857-0)

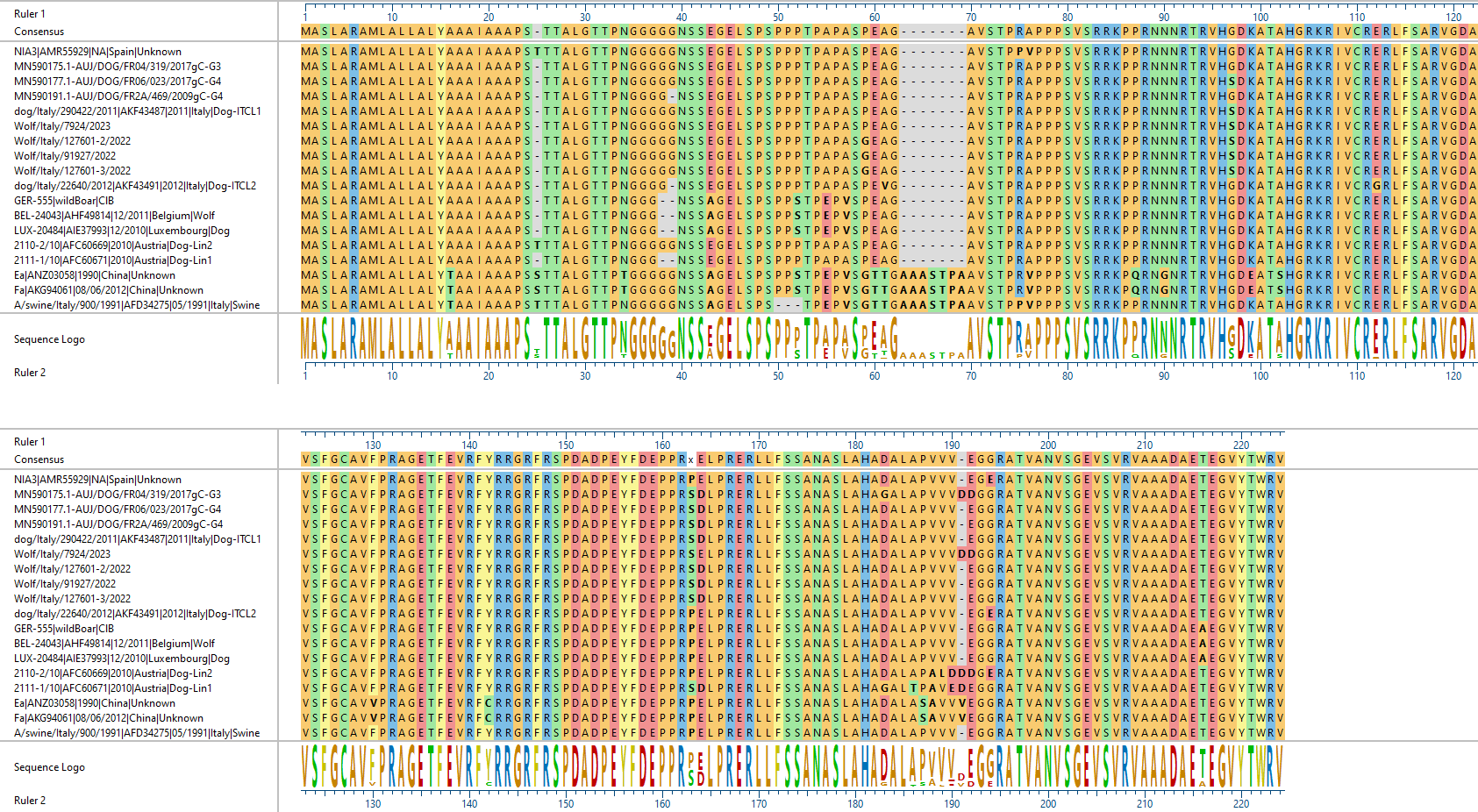

Supplement: Supplementary file 3 — Additional file 3: Fig. S1. Amino acid alignment of gC sequences of the Italian wolf samples that were compared with representative sequences of the Italian clade 1 and 2 and reference sequences obtained from wild boars, dogs and wolf originated from Europe and Asia. [file 12917_2023_3857_MOESM3_ESM.png]
